# Clinical Phenotype of HLA B*44 Patients in a Rheumatology Outpatient Clinic Favors Peripheral Arthropathies

**DOI:** 10.3390/jcm13185440

**Published:** 2024-09-13

**Authors:** Jure Aljinović, Daniela Šošo, Marin Petrić, Dijana Perković, Daniela Marasović Krstulović, Darko Kero, Ivanka Marinović

**Affiliations:** 1 Division of Physical Medicine and Rehabilitation with Rheumatology, University Hospital of Split, 21000 Split, Croatia; 2 University of Split School of Medicine, 21000 Split, Croatia; 3 University of Split, Department of Health Studies, 21000 Split, Croatia; 4 Department of Internal Medicine, Division of Rheumatology, Allergology and Clinical Immunology, University Hospital of Split, 21000 Split, Croatia; 5 Study Program of Dental Medicine, University of Split School of Medicine, 21000 Split, Croatia; darko.kero@mefst.hr

**Keywords:** haplotype, HLA-B*44, arthritis, pain, stiffness

## Abstract

**Objective:** The genetic background of HLA-B*27 in spondyloarthritis is known, and the search for another gene with similar role is ongoing. We wanted to investigate clinical presentations of HLA-B*44 patients in rheumatology practice. **Methods:** A cross-sectional retrospective study of 303 HLA-B*44 adult patients from the outpatient rheumatology clinic from 5/2018-5/2024. Clinical phenotype, confirmed or excluded rheumatic diagnosis, therapy used, and data on HLA A, B, and DR alleles inherited with B*44 were analyzed. **Results:** A female predominance of 2.79:1 was noted. A total of 150 [49.5%] patients were referred due to peripheral joint pain, 77 [25.4%] due to combined spine and peripheral joint pain or spine alone (57 [18.8%]). A total of 19 [6.3%] patients had no symptoms of the musculoskeletal system. Statistically significant peripheral joint affection was proved in females but not in males (*p* = 0.04). A total of 121 [40%] patients from B*44 group had established rheumatic disease, with the rest being excluded or under observation. The most common working diagnoses were polyarthritis (32 [10.5%]) and mono-oligoarthritis (14 [4.6%]). A second allele in addition to HLA B*44 showed a similar frequency to the general population. Patients with HLA B*44/44 and B*27/44 genotypes were at the most risk for having definitive rheumatic disease (>60%). Conventional synthetic disease-modifying anti-rheumatic drugs (DMARDs) were used in 38.6% of patients, non-steroidal anti-inflammatory drugs were used in 31.6% of patients, biologic DMARDs were used in 8.9% of patients, and corticosteroids were used in 7.3% of patients. **Conclusions:** The most common presentation in HLA-B*44 patients is peripheral joint affection. Most patients with HLA-B*27/44 and B*44/44 genotypes had definitive rheumatic disease. B*44 homozygosity or B*27/44 might be risk factors for arthritis development.

## 1. Introduction

Human leukocyte antigen B*44 (HLA B*44) is a split antigen of the broad antigen B*12 and is a sister type of B*45. HLA B*44 alleles are common in all ethnic populations in Europe at a frequency averaging from 9 to 15% [1], and is the third most common HLA B allele in the Croatian population, reported at 9.7% [2] and 9.3% incidence [3], after HLA B35 (13.4%) and HLA B51(5) (10.6%) [3]. HLA B*44 is subdivided into seven allelic forms that can only be discriminated by DNA typing. B*4402, B*4403, and B*4405 differ in one or two amino acids, which is enough to substantially alter T cell recognition [4]. Due to this, B*4402 and B*4403 have long been known as a ‘taboo mismatch’ in tissue transplantation [4].

The association of A2-B*4402 haplotypes with DRB1*0101, *0401, and *1301 was reported [5]. This is important from a rheumatology standpoint because shared epitopes of DRB1*0101, and 0401 were associated with a more aggressive disease course in rheumatoid arthritis (RA) [6,7]. Regarding other autoimmune diseases, a possible association of HLA B*44 and Cw*5 with Crohn’s disease was reported [8]. It was also found that a subset of patients with Crohn’s disease who develop ankylosing spondylitis (AS) have an increased incidence of the HLA B*27/B*44 allele compared to those without axial disease and healthy controls [9].

HLA B*44 patients with inflammatory bowel disease (IBD) also have an increased incidence of peripheral arthritis [10]. This peripheral affection is called type 2 arthropathy and affects 3–4% of patients with IBD. It is characterized by small joint involvement of the hands (metacarpophalangeal joints, proximal and distal interphalangeal joints) [11]. Unlike type 1 arthropathy which is oligoarticular, asymmetric, and affects large joints of the lower extremities, type 2 arthropathy is not associated with active bowel disease [11]. It is also possible that type 2 arthropathy forms a separate clinical entity due to different HLA associations and its overall different etiology [10].

HLA B*44 was found to be more frequent in some other diseases, like recurrent sinopulmonary infections (RESPI), common variable immune deficiency (CVID) [12], and vesicoureteral reflux (HLA A*9 and B*44) [13], whereas the reports on susceptibility of HLA B*44 patients to multiple sclerosis (MS), Behçet disease (BD) and Lichen sclerosus and atrophicus (LSA) are contradictory [14,15,16,17,18]. In contrast, a lower frequency of HLA B*44 was found in patients with rheumatic heart disease [19]. It might be possible that HLA B*44 does not affect the incidence of any disease. However, there is evidence that it can change the presentation of diseases, as is the case with IBD.

This paper tries to establish the clinical phenotype of HLA B*44 patients in our rheumatology outpatient clinic. We will analyze the incidence of peripheral and axial arthropathies and extra-articular presentations. Further comparison of subgroups regarding a second allele in addition to B*44 and its effect on clinical phenotype will be analyzed.

## 2. Materials and Methods

A cross-sectional retrospective analysis of data from HLA B*44-positive patients was performed on adult patients found in the medical electronic archives of the Department of Rheumatology and Clinical Immunology and the Department of Physical and Rehabilitation Medicine with Rheumatology, Clinical Hospital Centre Split in Croatia. Data were available from the time period from 1 May 2018 to 30 May 2024. The study was conducted in accordance with the Declaration of Helsinki, and was approved by the Ethical Committee of the University Hospital Split (520-03/24-01/25, 22 February 2024). All patient data were fully anonymized, and patients were coded with identities known only to the principal investigator.

The inclusion criterion for the study group was HLA B*44 positivity in patients who reported to the rheumatology outpatient clinic. Data on HLA A, B, Bw, Cw, and DR alleles inherited with B*44 were analyzed if available. Incidence of rheumatic diagnosis was noted, and all types of therapy. In our institution, all patients receive a medical report with a leading diagnosis classified by the International Classification of Diseases, Tenth Revision (ICD-10) that was further analyzed. The location of pain was noted and divided into four groups: (a) peripheral joint pain, (b) pain in the spine and hips, (c) significant pain in both spine and peripheral joints, and (d) no pain in the musculoskeletal (MSK) system.

The patient diagnosis was noted as definitive if it fulfilled the criteria for inflammatory rheumatic disease (AS based on the modified New York criteria, psoriatic arthritis (PsA) based on CASPAR criteria, ASAS classification criteria for axial spondyloarthritis (axSpA) and non-radiographic axSpA, EULAR/ACR 2020 for RA, and for the Polymyalgia Rheumatica 2012 EULAR/ACR Provisional Criteria…). Additionally, patients that did not fulfill classification criteria but had significant symptoms and received therapy with conventional synthetic disease-modifying anti-rheumatic drugs (csDMARD), such as methotrexate (MTX), leflunomide (LEF), sulfasalazine (SSZ), and hydroxychloroquine (HCQ), or biologic therapy (bDMARD) for more than three months were also regarded as being part of the definitive rheumatic diagnosis group.

For the working diagnosis group, we have selected diagnoses, such as polyarthritis, oligoarthritis, and monoarthritis in observation that do not fulfil diagnostic criteria and/or have been on DMARDs for under 3 months. After the diagnostic process, patients who were defined as having degenerative disease were allocated into the following group: excluded rheumatic disease. In this group, we have included several diagnoses, such as osteoarthritis of the hands, hip, knee, and spine and cervical and lumbar painful syndromes.

The patients were allocated to each group in agreement with three authors (J.A., I.M., D.Š.).

The clinical characteristics of patients with HLA B*44 homozygotes and HLA B*44 with other alleles comprising at least 5% (n ≥ 15) of the total cohort size were compared.

### Statistical Analysis

Statistical analysis was performed in Microsoft Office Excel 2016 32-bit (Microsoft Corporation, Redmond, WA, USA). Classical methods of descriptive statistics were used. Continuous data were presented as means with standard deviations (SD). Categorical variables were presented as a number of cases and percentage. The chi-square test was used to compare differences in clinical presentation, specific diagnosis, and confirmed or excluded rheumatic diagnosis between sexes. Also, to compare confirmed or excluded rheumatic diagnosis between groups divided by a second HLA B allele in addition to B44, chi-square post hoc analysis was performed using a pairwise binomial post hoc test with Bonferroni adjustment. The level of statistical significance was set at α = 0.05 (*p* < 0.05).

## 3. Results

In the study group of 303 HLA B*44-positive patients, a female predominance of 2.79:1 (223 [75.6%] females vs. 80 [24.4%] males) was noted.

The mean ages of females and males were 56.1 ± 13.5 years and 53.6 ± 14.9 years, respectively.

The leading symptom for referral to a rheumatologist was pain in peripheral joints in 150 [49.5%] of patients, followed by combined pain in the spine and peripheral joints in 76 [25.4%], spine alone in 57 [18.8%], and no symptoms of the MSK system in 19 [6.3%] of the patients (patients referred with abnormal laboratory testing findings, autoimmune hepatitis, recurrent uveitis, Raynaud phenomenon, etc.).

Statistical significance was found regarding disease presentation between sexes. In females, a clinical presentation that favored peripheral joint pain was more frequent, while males did not have differences between the incidence of peripheral joint pain and spinal pain (Table 1). The patients with both spinal and peripheral pain were allocated to the spinal pain group.

In total, around 212 [69.9%] of all patients had a rheumatic diagnosis confirmed or excluded, while 91 [30.1%] were under evaluation or did not fulfil the criteria for a rheumatic diagnosis (Table 1). No statistical significance was found in the occurrence of definitive rheumatic diagnosis and the definitive exclusion of rheumatic conditions between sexes. The patients who are still under evaluation were excluded from this analysis.

In Table 2, diagnoses are listed according to the ICD-10 classification. The most common definitive diagnoses are lumbosacral and cervical pain syndrome in 42 [13.9%] patients, followed by degenerative osteoarthritis in 13%, and the diagnosis of spondyloarthritis in 39 [12.9%] of the patients. The most common arthritis in this cohort was PsA in 30 [9.9%] patients, followed by seropositive and seronegative rheumatoid arthritis, with 21 [6.9%] patients each. The most common working diagnosis was polyarthritis with 32 [10.5%] and mono- or oligoarthritis with 14 [4.6%] patients.

**Figure 1:**
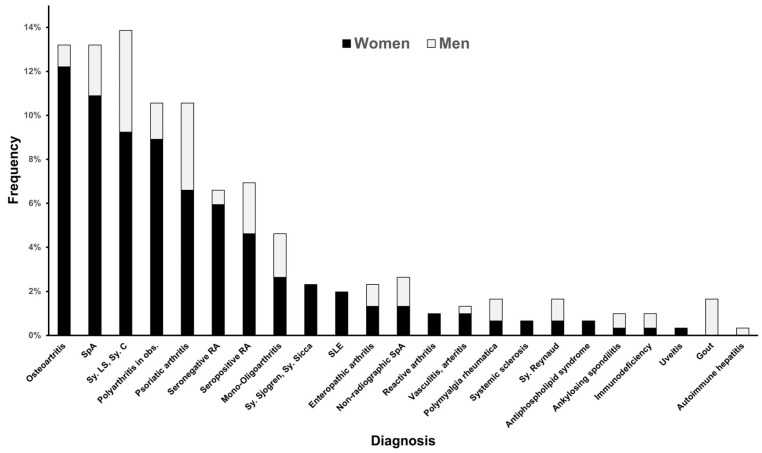
The frequency of diagnosis in HLA B*44 patients in males and females. Legend: SpA—spondyloarthritis; RA—rheumatoid arthritis; SLE—systemic lupus erythematosus; Sy. LS—syndroma lumbosacrale; Sy C—syndroma cervicale.

The frequency of different diagnoses was compared between sexes (Figure 1). The incidence of osteoarthritis was statistically significantly higher in women compared to men (chi-sq = 13.76; df 5; *p* = 0.017).

**Figure 2:**
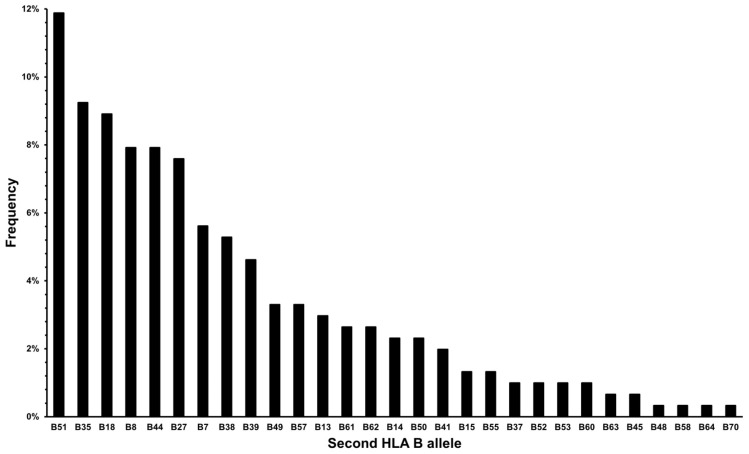
Second HLA B allele frequency in HLA B*44 patients.

The distribution of HLA B alleles other than the B*44 allele is presented in Figure 2.

The distribution of the HLA A, Bw, Cw and DR*B1 alleles is presented in Table 3.

Additional statistical analyses were performed on the HLA B alleles that accompany B*44 and appear in more than 5% frequency, namely: HLA B*5 (*51,*52), HLA B*16 (*38,*39), HLA B*35, HLA B*18, HLA B*12 (*44,*45), HLA B*27, HLA B*21 (*49,*50), and HLA B*7. The baseline data are presented in Table 4. A chi-square test with post hoc analysis was performed to determine whether there is a difference between confirmed and excluded rheumatic diagnosis. It was only significant for HLA B*27, which showed that rheumatic inflammatory disease is rarely excluded with the presence of this allele (z-crit −2.99, z value −5.21; chi-sq = 11.72, df 8). There was no statistically significant difference between groups regarding peripheral and axial presentation of the symptoms (chi-sq = 4.81, df 8, *p* = 0.771).

CsDMARDs were the most commonly used group of medication in the HLA B*44 cohort and were used in 117 [38.6%] patients in this order: MTX, HCQ, SSZ, and LEF (47 [15.5%], 34 [11.2%], 28 [9.2%], and 8 [2.6%], respectively). In 37 patients [31.6%], only non-steroidal anti-inflammatory drugs (NSAIDs) were used either on a regular or per-needed basis. A combination of tramadol and paracetamol was used by 15 [4.8%] patients, while 57 [18.8%] patients reported taking no medications. A total of 13 [4.3%] patients used corticosteroids either as a monotherapy or in combination with csDMARDs (9 [3.0%]) and with bDMARDs (9 [0.3%]).

In 27 [8.9%] patients, bDMARDs were used as a therapy option; they were used as a monotherapy in 16 patients [5.3%], in combination with csDMARD in 10 patients [3.3%], and with corticosteroids in 1 [0.3%] patient. Tumor necrosis factor (TNF) alpha inhibitors were used in 16 [5.3%] patients, interleukin (IL) 17 inhibitors were used in in 6 patients [1.9%], Janus Kinase 2 (JAK) inhibitors were used in 3 patients [0.9%], and IL6 and IL23 inhibitors in 1 [0.3%] case.

In 97 [32%] patients, this was not the first line of therapy; a total of 63 [20.8%] patients had 1 medication change, 25 [8.3%] had 2 changes, and in 9 [2.9%] patients, drugs were changed 3 times before this study. SSZ was changed in 21 [6.9%] patients, followed by MTX in 20 [6.6%] patients, HCQ in 14 patients [4.6%], LEF in 3 patients [0.9%], and TNF alpha inhibitors in 4 [1.3%] cases. NSAIDs are excluded from the therapy in 30 [9.9%] patients due to side effects.

## 4. Discussion

As we were trying to establish an SpA-like joint affection in HLA B*44-positive patients, we found that a significant majority of patients reported peripheral joint affection (around 75% of cases), with either peripheral joint affection only or combined with pain in the spine. Presentation affecting both spine and peripheral joints did not differ in frequency between groups, considering a second HLA B allele. The seven most common alleles from the Croatian population (n = 10,000) were found in this cohort with similar percentages, thus confirming that no other allele accumulation affected clinical presentation beside B*44 (HLA B*51 11.8% vs. 10.6%, B*35 9.2% vs. 13.4%, B*18 8.9% vs. 8.6%, B*44 8% vs. 9.7%, B*8 7.9% vs. 8%, B*27 7.6% vs. 6.3%, B*7 5.6% vs. 7.3%, cohort vs. control population, respectively) [2].

A significant percentage of HLA B*44-positive patients had established rheumatic disease (40% of cases), mostly SpA, PsA, and RA. We have found that the subgroups HLA B*27/44 and B*44/44 had the highest risk (more than 60%) for definitive rheumatic disease.

Although the most common clinical presentation of HLA B*44 patients was peripheral joint pain, statistical significance was proven in female patients but not in males. The reason for that could be a lower number of male patients, which needs to be increased to adequately determine the significance. This peripheral disease presentation with the involvement of metacarpophalangeal and proximal interphalangeal joints resembles the one from early or very early rheumatoid arthritis [20]. CD4+ T-helper lymphocytes are responsible for the initiation and prolongment of inflammation and autoantibody production in RA. They are predominant in synovial tissue [21]. Other T lymphocytes, such as Th1, Th17, Treg, and Th22 cells, contribute to psoriasis and PsA development [22]. The function of HLA B alleles, including HLA B*44 is to present antigenic peptides to CD8 positive cytotoxic lymphocytes, similar to how HLA B*27 has a role in spondyloarthritis [23]. We propose that joint affection in HLA B*44-positive patients is the result of the activation of both CD4 and CD8 positive cells. As this is a cross-sectional retrospective study, we did not have the opportunity to analyze the activity of these T cells. Since T cells are important in early arthritis models, we have researched the literature for potential hyperreactivity of T cells in B*44 individuals in other medical fields.

In gastroenterology, HLA B*27/44 was described in IBD with type 2 arthropathy [8,9,10,11], but we did not find an increased incidence of IBD, enteropathic arthritis, or reactive arthritis in these patients. Future studies should include a large cohort of patients referred to a gastroenterological outpatient clinic due to symptoms indicative of IBD, perform HLA typing, and follow up for musculoskeletal manifestations to prove a definitive role of B*44 in these conditions.

The immunologic role of B*44 is described in detail in transplantation. A strong autoimmune response that involved the T cell recognition process was suggested as a reason for the increased risk of acute graft-versus-host disease in B*44 patients who required hematopoietic stem cell transplantation (HSCT) [24], implicating a significant role of activated CD8 positive T cells [25]. However, oral mucositis as an HSCT complication is milder and shorter [26]. Even the difference between B44 alleles e.g., B*4402 and B*4403 can result in increased tissue rejection [4]. This still not fully understood T cell recognition hyper-reactivity could be the reason for the change in clinical presentations in HLA B*44 patients.

It was shown that B*44 positivity increases susceptibility to some infections, while for others it is protective. Slow progression of human immunodeficiency virus-positive patients to acquired immunodeficiency syndrome (AIDS) was described [27,28]. However, B*44 patients with developed AIDS were more susceptible to cytomegalovirus (CMV) retinitis and encephalitis due to low T cell immune responses to CMV [29], suggesting altered CD8-positive T cell activation responsible for viral immunity. The specific haplotype of HLA-A*2, -B*44, and -DR*4 was connected to CMV and Mycobacterium avium complex disease in AIDS patients [30]. Development of Stevens–Johnson syndrome with ocular involvement after the usage of NSAIDs and multi-ingredient cold medications was associated with HLA-A*0206 and HLA-B*4403 in the Japanese and with HLA-B*44:03 in Indian and Brazilian populations [31], possibly also involving T cell over-reactivity. A permissive role of HLA C*01 and B*44 towards SARS-CoV-2 infection was described [32] and an increased frequency of HLA B*44 was found in the patients with lung hydatid disease [33].

B*44 was found to have a protective effect in some diseases: a protective effect on developing dengue hemorrhagic fever was described [34], as well as decreased incidence in smear-positive pulmonary tuberculosis development [35,36]. The B*44 allele was overrepresented in milder forms of type Ia autoimmune lymphoproliferative syndrome [37].

B44 was also mentioned in oncology in the light of decreased T cell presentation. The loss of human leukocyte antigen class I (HLA-I) in tumors is a major escape mechanism from the T cell-mediated response [38]. In colorectal tumors, the most frequent loss of the HLA class I allele was HLA B*44, thus enabling tumors to develop [39]. In treatment with immune checkpoint inhibitors, the outcome was better in the HLA B*44 haplotype, with extended survival in two distinct cohorts, namely melanoma and non-small cell lung cancer patients [40]. It was hypothesized that therapeutic vaccines that target immunodominant HLA B*44 restricted neoantigens, expressed by melanoma, could be beneficial [40]. A connection between HLA B*44 and cancer was established in Mexican female patients between human papillomavirus type 16 infection with cervical cancer [41].

This research into the literature enabled us to hypothesize that increased T cell presentation could be a factor in a higher frequency of developing peripheral symptoms in this cohort, with similarity to early RA or peripheral joint affection of SpA. Also, this could partly explain a high percentage of peripheral arthritis patients even in the HLA B*27/*44 group where dominant spinal affection was expected.

Patients who have fulfilled classification criteria for some rheumatic disease or have been receiving csDMARD or bDMARD were considered to have rheumatic disease. As expected, HLA B*27 showed the highest percentage of patients with definitive rheumatic diagnosis (60.8%), but this was expected because HLA B*27 is included as a diagnostic criterion in relevant classifications. Interestingly, similar results were found for HLA B*44, with 60% of patients with a definitive diagnosis, which is the first time it has been described for B*44 homozygosity. These data were retrospectively collected from more than ten rheumatologists working in this institution and the team of three rheumatologists reached a consensus on the final diagnosis, thus limiting allocation bias.

In similar papers, the connection between the axial form of PsA and HLA B*8, B*38, B*39; th peripheral form of PsA and HLA B*18, B*27, B*38, and B*39; psoriasis and B*13, B*17, B*39, B*57, and DR*7, and AS (axial form) and B*13, B*27, B*40, B*47, and B*51 was established [42]. Also, SpA in HLA B*35 patients was described in the literature as a possible separate entity [43,44]. In this paper, all other alleles inherited together with B44 had less than 43% of definitive rheumatic diagnosis, which is substantially less than the B*44/44 and B*27/44 group.

Surely, a follow-up of these patients in two years period could be beneficial because it will show us whether the 20–40% of patients in each subgroup that are regarded as under observation will have started on DMARDs therapy and, thus, whether they can be regarded as having a definitive diagnosis.

### Limitations of the Study

Multicenter studies considering different protocols of diagnostics and treatment options, and basic studies of T cell presentation in symptomatic HLA B*44 patients could help us develop more informative opinions on whether HLA B*44 is a risk factor for the development of arthritis. The number of patients should be bigger especially when analyzing the effect of a second allele in addition to B*44 on clinical phenotype, and more male patients are needed. The bias of wrongly associating patients in different groups regarding clinical presentation, and confirmed, under observation, or excluded rheumatic diagnoses was addressed by assigning three rheumatologists to agree on this categorization. Some of the overlapping rheumatic and musculoskeletal conditions were missed because, in the available medical data, diagnoses were mostly encoded by dominant diagnosis. Due to the high heterogeneity of the diagnoses, it was not possible to analyze patients with less common diagnoses and overlapping rheumatic diseases.

## 5. Conclusions

The most common presentation in HLA B*44 patients is peripheral joint affection. When analyzing a second allele in addition to HLA B*44, we have found a distribution similar to general population. Subgroups HLA B*27/44 and B*44/44 were at the most risk for having definitive rheumatic disease (>60%). The most common arthritis was psoriatic arthritis, with 10% of patients, and seropositive and seronegative rheumatoid arthritis with 7% of patients each. The most common working diagnoses were polyarthritis (10.5%) and mono-oligoarthritis (4.6%). This peripheral disease presentation resembles the one from early arthritis and increased T cell presentation previously described in other fields of medicine could be responsible. More basic studies and clinical data are needed to assess whether B*44 homozygosity or B*27/44 are a risk factor for arthritis development.

Key Points:

- The most common presentation in HLA B*44 patients is peripheral joint affection (50%).
- The HLA B*44-positive patients had established rheumatic disease in 40% of cases; 32% were excluded and 28% were under observation.
- Phenotypes HLA B*27/44 and B*44/44 had definitive rheumatic disease in 60% of patients.

## Figures and Tables

**Table 1:** Clinical presentation of HLA B*44 patients.

Clinical Presentation	Females		Males		Total		Chi-sq *	*p*
	n	%	n	%	n	%		
1. Peripheral joints	119	53.4%	31	38.8%	150	49.5%	**5.03**	**0.025**
2. Spine	36	16.1%	21	26.2%	57	18.8%	3.94	0.050
3. Spine and joints	56	25.1%	21	26.2%	77	25.4%	0.04	0.841
4. No pain in the MSK system	12	5.4%	7	8.75%	19	6.3%	1.14	0.286
TOTAL	223	100%	80	100.00%	303	100%		
Established rheumatic diagnosis	**Females**		**Males**		**Total**		Chi-sq *	***p***
	n	%	n	%	n	%		
1. Yes	84	37.7%	38	47.5%	122	40.3%	2.37	0.124
2. Under investigation	67	30.0%	17	21.3%	84	27.7%	2.27	0.132
3. Excluded	72	32.3%	25	31.2%	97	32.0%	0.03	0.865
TOTAL	223	100%	80	100%	303	100%		

Legend: MSK—musculoskeletal; * Degrees of freedom (df = 1).

**Table 2:** Frequency and type of diagnosis in B*44 patients.

Diagnosis	ICD-10	N	%
Sy. LS, Sy. C	M54, M53	42	13.9%
Osteoarthritis	M15–19	40	13.2%
SpA	M46, M46.9	40	13.2%
Psoriatic arthritis	M07.0–M07.3	32	10.6%
Polyarthritis in observation	M13.0	32	10.6%
Seropositive RA	M05	21	6.9%
Seronegative RA	M06	20	6.6%
Monoarthritis, oligoarthritis	M13.1, M13.9	14	4.6%
Non-radiographic SpA	M46.1	8	2.6%
Enteropathic arthritis	M07.4–M07.6	7	2.3%
Sy. Sjogren, Sy. sicca	M35.0	7	2.3%
SLE	M32	6	1.9%
Gout	M10	5	1.7%
Polymyalgia rheumatica	M35.3	5	1.7%
Sy. Raynaud	I73.0	5	1.7%
Vasculitis, arteritis	M31	4	1.3%
Reactive arthritis	M02	3	0.9%
Ankylosing spondylitis	M45	3	0.9%
Immunodeficiency	D80–84	3	0.9%
Systemic sclerosis	M34, L94	2	0.7%
Antiphospholipid syndrome	D68.61	2	0.7%
Uveitis	H20	1	0.3%
Autoimmune hepatitis	K75.4	1	0.3%
TOTAL		303	100%

Legend: Sy. LS—syndroma lumbosacrale; Sy C—syndroma cervicale; SpA—spondyloarthritis; RA—rheumatoid arthritis; SLE—systemic lupus erythematosus.

**Table 3:** HLA A, Bw, Cw, and DRB1 allele distribution arranged by frequency in 303 patients (606 alleles) in HLA B*44 patients.

HLA A	n	%	Bw	n	%	Cw	n	%	DRB1*	n	%
**02**	211	34.8%	**4**	330	54.5%	**5**	120	19.8%	**07**	81	13.4%
**03**	70	11.6%	**6**	120	19.8%	**4**	74	12.2%	**13**	72	11.9%
**01**	46	7.6%	**5**	2	0.3%	**2**	63	10.4%	**16**	70	11.6%
**24**	42	6.9%	**x**	154	25.4%	**6**	36	5.9%	**04**	69	11.4%
**23**	34	5.6%				**1**	19	3.1%	**11**	65	10.7%
**11**	31	5.1%				**3**	14	2.3%	**01**	39	6.4%
**28**	24	3.9%				**7**	7	1.2%	**03**	38	6.3%
**32**	19	3.1%				**8**	3	0.5%	**15**	35	5.8%
**25**	16	2.6%				**x**	270	44.6%	**08**	16	2.6%
**26**	15	2.5%							**12**	16	2.6%
**31**	15	2.5%							**14**	5	0.8%
**29**	12	1.9%							**10**	3	0.5%
**33**	10	1.7%							**17**	2	0.3%
**30**	3	0.5%							**09**	1	0.2%
**68**	2	0.3%							**x**	94	15.5%
**09**	1	0.2%									
**39**	1	0.2%									
**x**	54	8.91%									

Legend: x—data missing or not collected.

**Table 4:** The incidence of diagnosis and clinical presentation in HLA B*44 patients regarding second HLA B allele.

B44+ Second HLA B allele		HLA-B5 (51.52)		HLA B16 (38.39)		HLA B35		HLA B18		HLA B12 (44.45)		HLA B8		HLA B27		HLA B21 (49.50)		HLA B7	
		n = 39	%	n = 30	%	n = 28	%	n = 27	%	n = 25	%	n = 24	%	n = 23	%	n = 17	%	n = 17	%
Rheumatic disease	1. Yes	14	35.9%	13	43.3%	12	42.9%	10	37.0%	15	60.0%	8	33.3%	14	60.9%	5	29.4%	5	29.4%
	2. In obs	12	30.8%	6	20.0%	8	28.6%	9	33.3%	3	12.0%	9	37.5%	8	34.8%	4	23.5%	7	41.2%
	3.No	13	33.3%	11	36.7%	8	28.6%	8	29.6%	7	28.0%	7	29.2%	1 *	4.3%	8	47.1%	5	29.4%
Clinical presentation	1. Periferal joints	20	51.3%	15	50.0%	15	53.6%	14	51.9%	14	56.0%	12	50.0%	9	39.1%	8	47.1%	12	70.6%
	2. Spine	4	10.3%	6	20.0%	3	10.7%	4	14.8%	3	12.0%	6	25.0%	5	21.7%	5	29.4%	2	11.8%
	3. Spine and periphery	13	33.3%	7	23.3%	7	25.0%	5	18.5%	7	28.0%	5	20.8%	9	39.1%	2	11.8%	3	17.6%
	4. No pain	2	5.1%	2	6.7%	3	10.7%	4	14.8%	1	4.0%	1	4.2%	0	0.0%	2	11.8%	0	0.0%
Final or working dg.	Osteoarthritis	7	17.9%	3	10.0%	6	21.4%	2	7.4%	2	8.0%	4	16.7%	1	4.3%	2	11.8%	2	11.8%
	Seronegative RA	6	15.4%	2	6.7%	2	7.1%	3	11.1%							2	11.8%		
	SpA	5	12.8%	3	10.0%	3	10.7%	1	3.7%	4	16.0%	4	16.7%	8	34.8%	2	11.8%	2	11.8%
	Psoriatic arthritis	4	10.3%	4	13.3%	3	10.7%	2	7.4%	3	12.0%	1	4.2%	3	13.0%	2	11.8%	5	29.4%
	Sy. LS, Sy.C	4	10.3%	6	20.0%	1	3.6%	5	18.5%	2	8.0%	3	12.5%			5	29.4%	2	11.8%
	Polyarthritis in observation	3	7.7%	4	13.3%	3	10.7%	5	18.5%	3	12.0%	3	12.5%	1	4.3%	1	5.9%	3	17.6%
	Seropositive RA	2	5.1%	1	3.3%	3	10.7%	3	11.1%	2	8.0%	1	4.2%	4	17.4%	1	5.9%	1	5.9%
	Enteropathic arthritis	2	5.1%	2	6.7%					1	4.0%								
	Monoarthritis, oligoarthritis	2	5.1%	2	6.7%	1	3.6%			2	8.0%					1	5.9%	1	5.9%
	Vasculitis, arteritis	1	2.6%							1	4.0%								
	Sy. Sjogren	1	2.6%					1	3.7%									1	5.9%
	Non-radiographic SpA	1	2.6%	1	3.3%	1	3.6%			1	4.0%			2	8.7%				
	SLE	1	2.6%			1	3.6%	1	3.7%	1	4.0%	2	8.3%						
	Reactive arthritis					1	3.6%							1	4.3%				
	Gout							1	3.7%	1	4.0%	1	4.2%						
	Ankylosing spondylitis													3	13.0%				
	Polymyalgia rheumatica									1	4.0%	1	4.2%						
	Immunodeficiency			1	3.3%	1	3.6%					1	4.2%						
	Uveitis					1	3.6%												
	Systemic sclerosis			1	3.3%	1	3.6%												
	Sy. Raynaud							1	3.7%	1	4.0%	2	8.3%			1	5.88%		
	Antiphospholipid syndrome							2	7.4%										
	Autoimmune hepatitis											1	4.2%						

Legend: SpA—spondyloarthritis; RA—rheumatoid arthritis; SLE—systemic lupus erythematosus; Sy. LS—syndroma lumbosacrale; Sy C—syndroma cervicale; * statistical significance chi-sq post hoc analysis.

## Data Availability

Anonymous data can be provided upon request.
